# Cancer Patients’ Experiences of Burden when Involved in Treatment Decision Making

**DOI:** 10.1177/0272989X251334979

**Published:** 2025-04-29

**Authors:** Fiorella L. Huijgens, Marij A. Hillen, Mette J. Huisinga, André N. Vis, Corinne N. Tillier, Hester S. A. Oldenburg, Gwen M. P. Diepenhorst, Inge Henselmans

**Affiliations:** Department of Medical Psychology, Amsterdam UMC, Amsterdam, Netherlands; Amsterdam Public Health, Quality of Care, Amsterdam, the Netherlands; Cancer Center Amsterdam, Cancer Treatment and Quality of Life, Amsterdam, the Netherlands; Department of Medical Psychology, Amsterdam UMC, Amsterdam, Netherlands; Amsterdam Public Health, Quality of Care, Amsterdam, the Netherlands; Cancer Center Amsterdam, Cancer Treatment and Quality of Life, Amsterdam, the Netherlands; Department of Medical Psychology, Amsterdam UMC, Amsterdam, Netherlands; Department of Urology, Amsterdam UMC, Amsterdam, Netherlands; Department of Urology, The Netherlands Cancer Institute, Amsterdam, Netherlands; Division of Surgical Oncology, The Netherlands Cancer Institute, Amsterdam, Netherlands; Division of Surgical Oncology, Amsterdam UMC, Amsterdam, Netherlands; Division of Surgical Oncology, Flevo hospital, Almere, Netherlands; Department of Medical Psychology, Amsterdam UMC, Amsterdam, Netherlands; Amsterdam Public Health, Quality of Care, Amsterdam, the Netherlands; Cancer Center Amsterdam, Cancer Treatment and Quality of Life, Amsterdam, the Netherlands

**Keywords:** decision making, patient-perceived burden, uncertainty, responsibility, patient-clinician communication

## Abstract

**Purpose:**

Patients are increasingly involved in decision making by their clinicians. Yet, there are concerns that involvement in decision making may cause emotional distress in patients. Little research has examined the nature of the burden experienced by patients confronted with a life-changing treatment decision. Therefore, we explored the nature and manifestations of burden experienced by patients with early-stage breast and prostate cancer regarding their involvement in decision making. We further aimed to identify patient-perceived causes and potential solutions for their experienced burden.

**Methods:**

We used semi-structured interviews to explore the perspectives of patients with early-stage breast and prostate cancer. Patients (*N* = 24) were eligible if they were diagnosed in the past 6 mo and reported some degree of burden regarding their involvement in decision making. Two researchers independently inductively coded the interviews using thematic analysis.

**Results:**

Patients described being burdened by the decision in various ways and at various moments in the decision-making process. Patients attributed their decision-related burden mainly to uncertainty, fear of making the wrong decision, insufficient guidance by their clinician, and feeling an overwhelming sense of responsibility for their treatment decision. Patients indicated various factors that mitigated their burden or facilitated decision making, including having sufficient time, the opportunity to discuss the choice with experts and/or family, and receiving advice or confirmation from family or the clinician.

**Conclusion:**

These findings suggest that decision-related burden could be caused by the uncertainty and anxiety patients experience and by a nonpreferred division of roles within the decision-making process.

**Implications:**

Accordingly, acknowledging patients’ feelings by discussing the presence of uncertainty and distress might normalize the burden for patients. Moreover, clinicians could explore and adjust to patients’ role preference in decision making and discuss what would facilitate the decision process for patients.

**Highlights:**

- Patients experience emotional, cognitive, and physical burden from their involvement in decision making.
- Some of the burden appears to result from the way clinicians involve patients in decision making.
- In addition to information about options, benefits, and harms, patients require active support in their decision-making process.
- Clinicians could aim to avoid overfocus on patient autonomy and instead establish authentic, shared decisions, with a role for some clinician control if needed.

## Introduction

Cancer patients increasingly face preference-sensitive treatment decisions,^1^ that is, decisions for which there is medically no optimal strategy, because the evidence for the treatment outcomes is lacking, treatment options have similar outcomes, or patients can be expected to differ in how they weigh the benefits and harms of the options.^2^ The ideal model of involving patients in such decisions is considered to be shared decision making (SDM).^3–5^ In SDM, patients and clinicians work together to make decisions about care that align with the best available medical evidence, the clinician’s expertise, and the values and preferences of the patient.^4,6,7^ A recent synthesis of SDM consultation models identified the following key components in the vast majority of models: creating choice awareness, describing treatment options, tailoring information, learning about the patient, discussing patient preferences, deliberating, and making or explicitly deferring the decision.^7^ SDM is generally considered morally justified, as patients’ autonomy and right to self-determination are supported.^4,8^ Empirical studies conclude that SDM improves patient outcomes, including satisfaction and, more tentatively, quality of life.^9–11^

However, some authors have raised concerns that the focus on patient autonomy may overlook patients’ vulnerability, especially shortly after diagnosis.^3,12–18^ Imposing decision-making responsibility on patients when they are psychologically vulnerable in uncertain situations may lead to emotional distress and conflict.^8,19–23^ Few studies have specifically examined the potential burden of involving patients in decision making,^24,25^ nor have they examined to what extent such burden depends on the way the components of SDM are applied by clinicians. Studies show that some men with prostate cancer experience distress due to the uncertainty surrounding the decision, pointing to a lack of clinician guidance.^19,26–28^ Similarly, women facing decisions regarding breast cancer surgery sometimes express concern and distress about making the right choice.^29^

Negative experiences around decision making may also arise when expectations or preferences regarding decisional roles are misaligned between physicians and patients. Notably, some but not all SDM models’ “determining roles in the decision making process” are included.^7^ Cancer patients who prefer a limited role in their own care process may particularly experience distress when these preferences are not sufficiently taken into account in the SDM process.^30,31^ Also, patients who expect a more family-centered decision-making process instead of a patient-centered approach may experience distress when the family is insufficiently involved by the clinician. This misalignment may especially be the case with Western clinicians and patients with non-Western cultural backgrounds, in particular when language or cultural barriers are present.^32^

Currently, we lack empirical evidence and in-depth understanding of patients’ perceived burden when involved in decision making. While existing literature offers hypotheses about its causes and solutions, little research has been purposefully initiated to investigate patients’ perspectives on their perceived burden of being faced with a treatment choice. Such research is needed to understand what kind of support could be offered to patients so that patients’ decision-related distress can be mitigated, optimizing the SDM process. To address this gap, we explored the nature and manifestations of burden experienced by patients with early-stage breast and prostate cancer regarding their involvement in decision making. We further aimed to identify patient-perceived causes and potential solutions for their experienced burden.

## Methods

### Design

A qualitative study using semi-structured one-on-one interviews with patients was conducted. These interviews generated in-depth personal accounts of the decision-making process. The consolidated criteria for reporting qualitative research (COREQ) were used to ensure complete methodological reporting (Appendix 1).^33^

### Sample and Recruitment

Participants were patients who had been diagnosed with early-stage breast cancer (tumor size <5 cm and ≤3 positive lymph nodes) or early-stage prostate cancer (no metastases outside of the prostate gland) in the past 6 mo. Patients were eligible if they were involved in a treatment choice (either recently or currently) by their clinician and self-reported some degree of burden regarding their involvement in decision making on a screening questionnaire (Appendix 2).

We chose to include early-stage breast and prostate cancer patients as these patients are relatively likely to be confronted with preference-sensitive treatment choices.^1^ In the Netherlands, SDM implementation in cancer care is increasingly promoted and embedded in the care for early-stage breast and prostate cancer patients.^34–36^

Participants were sampled purposefully, to ensure variation regarding potentially relevant characteristics,^37^ that is, their self-reported level of experienced burden, age, tumor type, type of treatment decision, and migration background. To further ensure a diverse sample, patients were recruited via 3 different ways: 1) via clinicians in 3 Dutch participating hospitals, 2) via 2 Dutch cancer patient associations (1 breast and 1 prostate), and 3) via a Dutch advocacy organization group for patients and their families with a migration background (Stichting Gezondheid Allochtonen Nederland). Interested patients completed the screening questionnaire digitally or verbally via telephone.

Eligible patients were informed about the study by the researchers, and if they provided initial oral consent, an appointment was made for an interview at the patients’ preferred location (home, hospital, or online via Microsoft Teams). At the time of the interview, patients signed formal informed consent. The total sample size was based on data saturation, meaning that recruitment continued until informational redundancy was achieved (i.e., when no new themes arose in 3 subsequent interviews).^38,39^

### Data Collection

Interviews were conducted by 4 researchers between October 2022 and February 2024. F.L.H. (MSc, background in health sciences and management) and M.J.H (MSc, psychologist), both trained in qualitative interviewing skills, conducted interviews with Dutch-speaking patients. Two care consultants with a non-Western background conducted interviews with Turkish-, Arabic-, and Berber-speaking patients. A semi-structured interviewing guide (Appendix 3) was used, based on the research questions (Table 1). The interview guide was pilot tested with 1 former breast cancer patient. Interviewers kept a reflexive journal capturing their thoughts before, during, and after the interview, to be aware of their own possible bias/assumptions. The interview guide was adapted and discussed with the core project group throughout the data collection based on emerging findings and the reflexive journals. The main changes in the interview guide were, for instance, focusing more on experiences and feelings rather than evaluations of care and focusing more on the choice process instead of care in general. Interviews were audio-recorded with participants’ permission and lasted about 1 h.

**Table 1:** Research Questions

Research questions
1.How do patients reflect on the manner and extent in which they were involved in decision making about their treatment? 2. How did patients’ involvement affected them positively and negatively? 3. What do patients believe could reduce the negative effects of their involvement?

### Data Analysis

**Figure 1:**
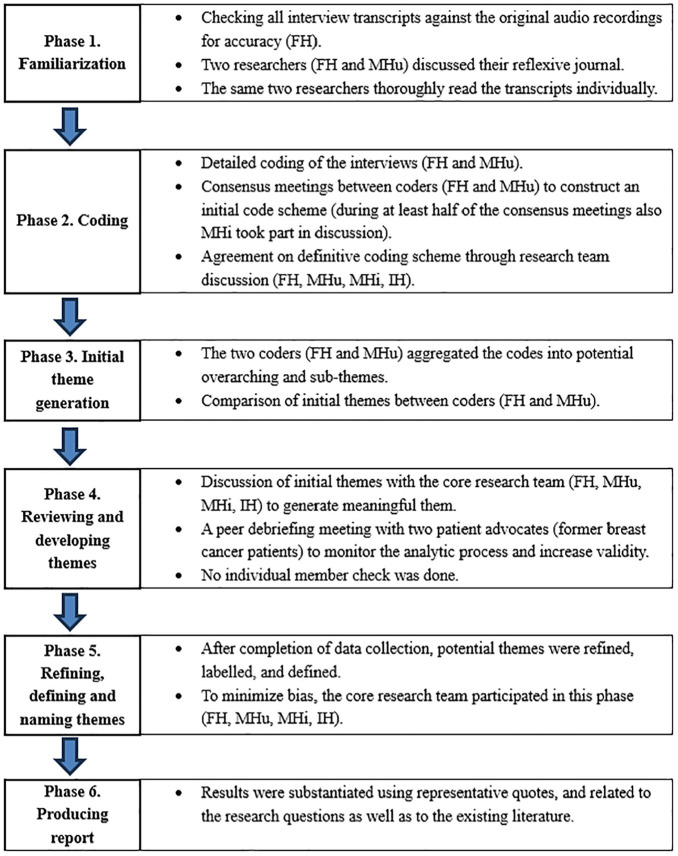
Six phases of reflexive thematic analysis as described by Braun and Clarke.^40,41^

Analysis was based on the 6 phases of reflexive thematic analysis described by Braun and Clarke,^40,41^ as illustrated in Figure 1. Analysis was mainly inductive, that is, not originating from existing theoretical frameworks but openly mapping experiences.^42^ Coding and analysis were performed with MAXQDA 2020 (Verbi software, Marburg, Germany).

## Results

### Sample Characteristics

In total, 50 patients completed the screening questionnaire, of whom 25 patients did not meet the inclusion criteria. Of those 25 patients, 6 were diagnosed longer than 6 mo ago, 2 patients did not experience a choice, 13 patients did not experience burden regarding their involvement, and 4 patients did not want to participate in the interview after completion of the questionnaire. Eventually, 24 patients were interviewed as 1 patient canceled the interview due to illness. Breast cancer patients (*n* = 13) faced choices about type of surgery (e.g., breast conserving v. mastectomy), type of breast reconstruction, radiotherapy, hormonal therapy, and/or chemotherapy. Patients with prostate cancer (*n* = 11) faced choices between active monitoring, surgery, radiotherapy, and hormonal therapy. See Table 2 for participant characteristics.

**Table 2:** Participant Characteristics

Characteristic	Breast Cancer (*n* = 13)	Prostate Cancer (*n* = 11)
Age, y		
<60	7	1
60–74	6	8
>75	0	2
Education^a^		
High	7	9
Medium	4	1
Low	1	1
Unknown	1	0
Degree of burden		
High	2	2
Medium	8	6
Low	3	3
Migration background		
Western	10	10
Non-Western	3	1

a Low = secondary education; middle = senior general secondary education, secondary vocational education, and preuniversity education; high = college or university.

### How Do Patients Experience Burden?

Patients reported experiencing the treatment choice they were facing as a dilemma, although they varied in how difficult the choice was for them, ranging from somewhat to very difficult. Some mentioned that it would have been easier if the clinician had decided for them or if they would not have been presented with a choice at all (Table 3, Q1).

**Table 3:** Illustrative Quotes in Relation to Research Questions

Research Question 1: How do patients reflect on the manner and extent in which they were involved in decision making about their treatment?	
Q1, Breast	“So I knew that choice was coming, but I actually hoped the entire time that—and I said as much to my mother—I hope they will make that choice. Because I don’t know if I can make that choice, so I hope that they make it.”
Q3, Breast	“You know, You just don’t expect it [a choice]. Because you go there [to the consultation], you have an intake meeting, right? You discuss a bit about what happened and how. And then, eventually, you’ve had your entire discussion. But—and then there was a but—and then comes the whole choice. Then I thought: ‘Well, huh?’ Totally unexpected.”
Q10, Breast	“I think it is quite much to have to decide for yourself, as a patient. I actually think you should say: ‘This is my advice.’ And, at the same time, make it explicitly clear that you can talk about it, that it is open for discussion, and that you can deviate from it. But yes, you do withhold advice from the patient. I actually find that very wrong. That’s going way too far.”
Q11, Breast	“At that time, in those 2 weeks, I was angry sometimes, you know. I thought that: ‘Well, this is not something that I can do.’ You can’t just let people decide. . . . Of course, there are consequences to your decision, and of course you yourself are responsible for that. And if the doctor decides, then the doctor is responsible for it.”
Q12, Breast	“I found it very difficult that they couldn’t help you with that choice. . . . Yes, what you decide—yes, that’s good. But then, I also asked: ‘What is wise, what’s the best thing to do then?’ But yes, they can’t help you with that.”
Research Question 2: How did patients’ involvement affected them positively and negatively?	
Q2, Prostate	“They said that it was my choice to determine what I really wanted in my situation. . . . I thought a lot about my choice and what I want. I thought a lot and I was afraid to make my own choice.”
Q4, Prostate	“Actually, really stupid. When I left there [from the consultation at the hospital] that first Friday, I was like: ‘Well. I’m not going to do it [hormone therapy].’ And eventually, during the week, I just became more and more unsure.”
Q5, Prostate	“I am now—I am now, of course, still concerned with: ‘Gosh, did I do it right?’ I’m now in the process of recovery. And, um, then comes: ‘Gosh, what am I going to face and what if I had decided differently?’ Yeah, that remains. Yeah.”
Q6, Prostate	“At some point—at least for me—at some point I do know that, okay, er, yeah, this is enough, you know, enough inside processing to be able to make a choice. And yeah, that was a restless time. But yeah, that time is sometimes also necessary. I know that too. . . . That it doesn’t feel quite comfortable or that it doesn’t feel quite pleasant. But yeah, it is what it is. Yeah, yeah, yeah. And if you asked this question while I was still in the middle of all that, it would have been harder to answer.”
Q7, Breast	“I must say that, during the weeks, I actually became more and more restless. I was thinking that: ‘Well, actually, my gut is saying no. No chemo.’ But I just want to find evidence for that ‘no,’ so to speak. I want to find some kind of basis that ‘no’ is the correct answer. I found that very difficult, that you actually have very little information about it, so to speak.”
Q8, Breast	“. . . Is it worth unleashing so much harm on your body for something that may not even happen at all? So I find that calculation of probability very difficult in this whole story. I find that very difficult about research, anyway. That calculation of probability. Yeah, you cannot actually base your choice on that. You have to decide based on your gut feeling, you have to base a gut choice on something scientific, on cold, hard numbers.”
Q9, Prostate	“But then you get these side effects, which are well-explained by all kinds of documentation on the internet and also in pretty pamphlets that you are given. But you see, a body, not every body is equal. So what’s going to happen to you? And now, of course, the choice becomes more difficult.”
Q13, Breast	“The big unknown for me was simply hoping that I wouldn’t regret it. Um. I’m a person—I’m always busy outdoors. I’d regret not being able to do those things later because I had to have a breast [reconstruction] immediately.”
Research Question 3: What do patients believe could reduce the negative effects of their involvement?	
Q14, Prostate	“Yes indeed, and that is my choice. Yes, that was certainly right. One has nothing else to do but trust in God. There is nobody else above God to trust in.”
Q15, Prostate	“I was glad that I said: ‘what a difficult choice all of a sudden.’ And then the nurse specialist said that having a choice was actually a luxury problem. So they tried to lighten the situation a bit.”

#### Emotional, cognitive, and physical manifestations of burden

Patients reported different ways in which the burden of choice would manifest. Manifestations could be 1) emotional, such as crying, fear, agitation, or being angry; 2) cognitive, such as constantly changing ideas, worry, grinding over the choice, being confused, or being uncertain; or 3) physical, such as sleep problems. These manifestations could go hand in hand (Table 3, Q2).

Patients could feel burdened at different moments throughout the process. This included the moment they were faced with the choice, while being “at home” after being faced with the choice, and/or the period afterward. The burden experienced during the moment they were faced with the choice mainly consisted of confusion. They felt confused when presented with a different choice than expected (as they had searched the internet for information or heard stories from others about specific treatments) or when unexpectedly presented with a choice instead of a treatment advice/proposal (Table 3, Q3). Others experienced burden once they had returned home after the consultation in which they were presented with a choice. These patients explained that during the consultation they felt that they had come to a decision, but upon leaving the hospital or arriving at home, doubts arose about what the “right” choice would be (Table 3, Q4).

#### Reflections after decision making range from satisfaction to doubts

In the period after the decision-making process, patients were overall satisfied with having been offered a choice despite the experienced decisional burden. However, they varied in how they reflected on the decision-making process. These views ranged from feeling relieved at the end of the decision-making process that the choice was eventually made to experiencing lingering doubts. Also, some patients believed going through this process, with all the difficulties it entailed, was inevitable to arrive at the “right” choice. This difference is reflected in the quotes of 2 prostate cancer patients (Table 3, Q5 and Q6).

**Figure 2:**
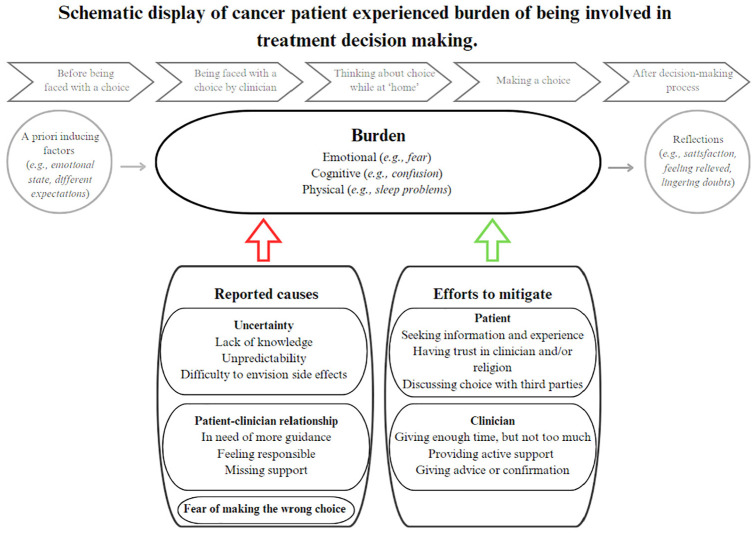
Schematic display of cancer patient–experienced burden of being involved in treatment decision making.

Figure 2 shows the schematic display of cancer patient–experienced burden of being involved in treatment decision making.

### What Induces Patients’ Experience of Burden?

Patients mentioned several factors that induced their experienced burden of involvement in treatment choices.

#### Uncertainty due to lack of knowledge and unpredictability

First, they explained how various types of uncertainty caused burden. This included an awareness of their own *lack of medical knowledge*. Making a choice based only on feelings instead on medical information felt wrong (Table 3, Q7). Another type of uncertainty was that patients felt burdened by the *unpredictability* of survival benefit, treatment effects, and side effects. Patients indicated it was difficult to decide based on statistical data, without specific predictions regarding their individual situation (Table 3, Q8). Especially, when the clinician indicated that treatment efficacy was predicted to be in the “gray” area of beneficence (i.e., medically no preference). Lastly, patients felt uncertainty because they experienced *difficulty envisioning side effects.* Some patients found it difficult to predict how severe the impact of side effects would be on their health, daily life, and/or relationships, complicating their decisional process (Table 3, Q9). Patients’ uncertainty was influenced by other people’s experiences and opinions. Uncertainty could be exacerbated by others, as patients heard/read various stories with conflicting outcomes from different people or because people suggested a different treatment option than the patient had in mind. Uncertainty could also be alleviated by others, as patients’ treatment choice/preference would be supported by others.

#### Misalignment in role preferences regarding decision making

A second cause of burden identified by patients was certain aspects of the patient–clinician relationship. Experiences with the division of roles varied between patients, with some patients expressing dissatisfaction with their role within the decision-making process. For example, one experience was that clinicians could have given more guidance and been more directive on what option to choose (Table 3, Q10). Another experience was the wish to not have full responsibility during the decision-making process (Table 3, Q11). Others also would have liked support from their clinicians in the decision making but seemed to believe a clinician is unable to provide such support (Table 3, Q12).

#### Fear of making the wrong choice

A third cause of burden patients mentioned was fear of making the wrong choice. This fear resulted from the irreversibility of the choice, worry about cancer recurrence, and anticipated regret. Patients’ anticipated regret was reflected in their comments suggesting they would blame themselves if the future outcome was not as desired (Table 3, Q13).

### How to mitigate the burden?

#### Patient efforts: gathering information and trusting the clinician

Ways in which patients dealt with being faced with a choice were various and included seeking information and experiences on the internet and discussing the choice with third parties, for instance, with family and friends but also with other clinicians or their general practitioner. Another way patients dealt with a choice was related to having trust. Some patients seemed to reassure themselves by trusting in the knowledge and experience of their clinician. Especially patients with a migration background mentioned reassuring themselves by trusting Allah. They felt that it was up to Allah whether they would be cured (Table 3, Q14).

#### Clinician efforts: offering time, support, and advice

Patients indicated 3 ways in which clinicians had helped them in making a choice and mitigated the burden they experienced. First, patients needed enough time, but not too much, to think about which choice to make. If given too much time, the choice became harder as doubts reemerged. Second, clinicians who provided active support in constructing a treatment preference also alleviated patient burden. For example, clinicians offered a new perspective by presenting the choice in a different way (Table 3, Q15), or helped to weigh the options against the patient’s preferences. Third, it helped patients when their clinician gave advice on the options or confirmed that the patient made a “good” choice. This gave the patient peace of mind and helped them put their doubts aside. Also, some patients who did not get any confirmation from their clinician expressed their desire for it to be sure they made the right choice.

## Discussion

We gathered detailed accounts of cancer patients’ experienced burden during their involvement in treatment decision making and their views on its causes and solutions. While patients valued being involved in treatment choices, they reported emotional (fear, anger), cognitive (confusion, procrastination), and physical (sleep issues) burden at different stages of the process. Patients attributed their burden to uncertainty, a felt decisional responsibility, fear of making the wrong decision, and insufficient guidance from clinicians.

Burden was experienced at various moments throughout the decision-making process. First, during consultations, several patients reported being unprepared for the presentation of choice, which required a mind shift. This suggests that not all patients expect to be involved in a treatment choice, even when SDM has been advocated in public campaigns in the Netherlands,^43^ and most patients nowadays indicate wanting to be involved in medical decisions.^44^ Therefore, clinicians might be aware that participation in decision making is not always evident for every patient. Even when patients anticipate having a choice, they may not be fully prepared for SDM, for example, because they lack medical knowledge or have difficulty understanding the information provided.^45–47^ Patients in our sample indicated that a lack of medical knowledge was related to experiencing distress. Reduced health literacy could further hinder engagement in SDM.^48^ Second, burden could be experienced after consultations, when patients were instructed to (re)consider their decision at home. People have different decision styles to cope with a decision, some of which are more likely to induce burden and a sense of abandonment when independently considering a choice.^49–51^ For example, people who rely on others or are avoidant in making decisions may feel unable to make effective decisions themselves or fear taking on responsibility.

Part of the reported burden may be inevitable, as cancer treatment decision making inherently involves uncertainty and stakes are usually high.^3,4,6,8,52^ Clinicians will involve patients in decision making only when, from a medical point of view, there are multiple realistic options. To make a decision that aligns with patients’ values and preferences, clinicians have no other choice than to involve the patient in the decision-making process. Uncertainty about the best course of action may create or add to the patient’s awareness of vulnerability after a cancer diagnosis.^8^ In our study, such vulnerability manifested in the unease patients felt regarding responsibility and their fear of making the wrong choice.

Our findings, however, also show that part of the burden may be related to how patients are involved in decision making by their clinician. Some patients felt highly responsible, were in need of more guidance, and missed support in the decision-making process. These experiences seem misaligned with SDM as intended, instead aligning with practices of informed decision making.^53,54^ The informative decision-making model assumes that patient’s values are well defined and known, limiting the physician’s role to providing relevant factual information and executing the patient’s choice.^54^ In other words, the conception of patient autonomy in informed decision making is patient control and patient responsibility over decision making,^53^ while in SDM, clinician guidance is important. Our results show that such guidance may sometimes fall short, as patients expressed a need for more direction. Some indicated this would help them relieve decisional burden by being able to trust in their clinicians’ expertise. Similarly, previous empirical evidence reiterates that patients’ feeling of engagement and autonomy often go hand in hand with the wish to be cared for by their clinicians.^55^ Thus, while clinicians may sometimes approach patients as autonomous, rational decision makers, some patients may require more guidance in the decision-making process. The challenge for clinicians is to balance providing patient autonomy with giving sufficient support.^55,56^

When it comes to patients’ wish to trust in clinician expertise, clinicians’ main concern might also be to strike the right balance. On one hand, our results show trust is needed to offer guidance and potentially lower patients’ decisional burden. On the other hand, excessive trust may cause dependency on the clinician^8,47^ and less active participation in the decision-making process.^47^ This can become especially problematic when doctors are unaware how such dependency may reduce patients’ autonomous capacity.^8^ Ultimately, clinicians may keep in mind that SDM prioritizes that choices align with patient’s needs and preferences, regardless of who eventually makes the final decision.

### Conceptual and Practical Implications

**Figure 3:**
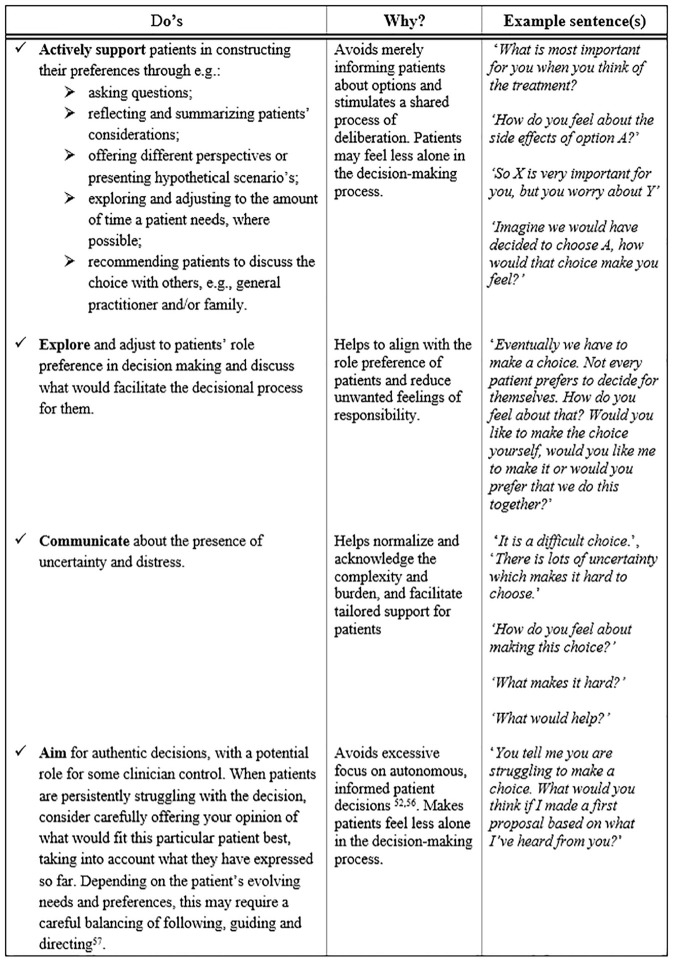
Practical implications for clinicians regarding shared decision making.

In recent SDM models, the emphasis has shifted away from clinicians providing recommendations and/or sharing their professional preferences.^7^ This movement signals a drive to enhance patients’ autonomy and right to self-determination. However, the current results highlight the potential unintended negative consequences for the patient if clinicians fully refrain from providing their input. Our findings emphasize the importance of clinicians providing guidance and support in preventing and mitigating patients’ decisional burden. Further, our results also show the importance of explicitly addressing and agreeing on roles in decision making to prevent patients from feeling the responsibility for a decision is imposed on them. Thus, the current study underscores that conceptualizations of SDM should include elements pertaining to the determination of preferred roles and leave room for some physician control in decision making. Figure 3 lists several practical recommendations for clinicians in the context of mitigating a patient’s burden regarding decision making.^53,57,58^

### Strengths and Limitations

A strength of our approach is that we used researcher triangulation, which increased the validity of our findings and added depth to the phenomenon of interest.^59,60^ Limitations are, first, that most patients were interviewed after completing the decision-making process. Thus, their reflections may have been affected by the passage of time. Second, despite our inclusion of 4 patients (17% of our sample) with a non-Western migration background, our sample consisted largely of highly educated, native Dutch people. Additional perspectives of lower educated patients and patients with a non-Western background are needed, as experienced burden may be affected by language barriers and cultural differences.^32^ Finally, in interpreting the results, readers should be aware that we included only patients with self-reported burden and only in the context of curative breast and prostate cancer. Therefore, these findings may not generalize to other populations. We did not focus on potential differences in patients’ experiences of burden between various settings. Future research could explore these, for example, between cancer types and different types of decisions associated with them and/or curative versus palliative setting. In addition, it would be interesting to compare differences in perspectives between patients who experienced burden and those who did not, to gain more insights into predictors of burden.

## Conclusion

This study provides important insights into cancer patients’ experiences of burden when being involved in treatment decision making. Experienced burden varied in nature and manifested at various moments throughout the decision-making process. The results suggest that the burden can be partly explained by how patients are involved in treatment decision making, highlighting the importance of active support and guidance from clinicians in the decision-making process.

## Supplemental Material

sj-docx-1-mdm-10.1177_0272989X251334979 – Supplemental material for Cancer Patients’ Experiences of Burden when Involved in Treatment Decision Making Supplemental material, sj-docx-1-mdm-10.1177_0272989X251334979 for Cancer Patients’ Experiences of Burden when Involved in Treatment Decision Making by Fiorella L. Huijgens, Marij A. Hillen, Mette J. Huisinga, André N. Vis, Corinne N. Tillier, Hester S. A. Oldenburg, Gwen M. P. Diepenhorst and Inge Henselmans in Medical Decision Making

## References

[1] Wennberg JE . Preference-sensitive care: a Dartmouth Atlas project topic brief. Lebanon (NH): The Dartmouth Institute for Health Policy and Clinical Practice; 2007. 36441855

[2] Gärtner FR Portielje JE Langendam M , et al. Role of patient preferences in clinical practice guidelines: a multiple methods study using guidelines from oncology as a case. BMJ Open. 2019;9:e032483. DOI: 10.1136/bmjopen-2019-032483 PMC6924854 31811009

[3] Barry MJ Edgman-Levitan S . Shared decision making—the pinnacle of patient-centered care. New Engl J Med. 2012;366:780–1. DOI: 10.1056/NEJMp1109283 22375967

[4] Stiggelbout AM Pieterse A De Haes J . Shared decision making: concepts, evidence, and practice. Patient Educ Couns. 2015;98:1172–9. 10.1016/j.pec.2015.06.022 26215573

[5] van der Horst DEM Garvelink MM Bos WJW Stiggelbout AM Pieterse AH . For which decisions is shared decision making considered appropriate? A systematic review. Patient Educ Couns. 2023;106:3–16. DOI: 10.1016/j.pec.2022.09.015 36220675

[6] Charles C Gafni A Whelan T . Shared decision-making in the medical encounter: what does it mean? (or it takes at least two to tango). Soc Sci Med. 1997;44:681–92. DOI: 10.1016/s0277-9536(96)00221-3 9032835

[7] Bomhof-Roordink H Gartner FR Stiggelbout AM Pieterse AH . Key components of shared decision making models: a systematic review. BMJ Open. 2019;9:e031763. DOI: 10.1136/bmjopen-2019-031763 PMC6937101 31852700

[8] Gulbrandsen P Clayman ML Beach MC , et al. Shared decision-making as an existential journey: aiming for restored autonomous capacity. Patient Educ Couns. 2016;99:1505–10. 10.1016/j.pec.2016.07.014 27460801

[9] Shay LA Lafata JE . Where is the evidence? A systematic review of shared decision making and patient outcomes. Med Decis Making. 2015;35:114–31. DOI: 10.1177/0272989X14551638 PMC4270851 25351843

[10] Kehl KL Landrum MB Arora NK , et al. Association of actual and preferred decision roles with patient-reported quality of care: shared decision making in cancer care. JAMA Oncol. 2015;1:50–8. DOI: 10.1001/jamaoncol.2014.112 PMC4937185 26182303

[11] Kashaf MS McGill E . Does shared decision making in cancer treatment improve quality of life? A systematic literature review. Med Decis Making. 2015;35:1037–48. DOI: 10.1177/0272989x15598529 26246515

[12] Olthuis G Leget C Grypdonck M . Why shared decision making is not good enough: lessons from patients. J Med Ethics. 2014;40:493–5. 10.1136/medethics-2012-101215 23660518

[13] Fisher KA Tan ASL Matlock DD Saver B Mazor KM Pieterse AH . Keeping the patient in the center: common challenges in the practice of shared decision making. Patient Educ Couns. 2018;101:2195–201. DOI: 10.1016/j.pec.2018.08.007 PMC6376968 30144968

[14] Thorne S Oliffe JL Stajduhar KI . Communicating shared decision-making: cancer patient perspectives. Patient Educ Couns. 2013;90:291–6. DOI: 10.1016/j.pec.2012.02.018 22464665

[15] Rutten LJ Arora NK Bakos AD Aziz N Rowland J . Information needs and sources of information among cancer patients: a systematic review of research (1980–2003). Patient Educ Couns. 2005;57:250–61. DOI: 10.1016/j.pec.2004.06.006 15893206

[16] de Haes H . Dilemmas in patient centeredness and shared decision making: a case for vulnerability. Patient Educ Couns. 2006;62:291–8. 10.1016/j.pec.2006.06.012 16859860

[17] Epstein RM . Whole mind and shared mind in clinical decision-making. Patient Educ Couns. 2013;90:200–6. 10.1016/j.pec.2012.06.035 22884938

[18] Clayman ML Gulbrandsen P Morris MA . A patient in the clinic; a person in the world. Why shared decision making needs to center on the person rather than the medical encounter. Patient Educ Couns. 2017;100:600–4. 10.1016/j.pec.2016.10.016 27780646

[19] Henrikson NB Ellis WJ Berry DL . “It’s not like I can change my mind later”: reversibility and decision timing in prostate cancer treatment decision-making. Patient Educ Couns. 2009;77:302–7. DOI: 10.1016/j.pec.2009.03.017 PMC3509197 19386460

[20] Kristvik E . For whom and for what. Exploring the question of ‘informed consent in treatment decision making processes. Med Antropol. 2011;23:9–43.

[21] Politi MC Lewis CL Frosch DL . Supporting shared decisions when clinical evidence is low. Med Care Res Rev. 2013;70:113s–28s. DOI: 10.1177/1077558712458456 23124616

[22] Berger Z . Navigating the unknown: shared decision-making in the face of uncertainty. J Gen Intern Med. 2015;30:675–8. DOI: 10.1007/s11606-014-3074-8 PMC4395589 25536912

[23] Vickers AJ . Decisional conflict, regret, and the burden of rational decision making. Med Decis Making. 2017;37:3–5. DOI: 10.1177/0272989x16657544 27899744 PMC5137809

[24] van de Water LF Bos–van den Hoek DW Kuijper SC , et al. Potential adverse outcomes of shared decision making about palliative cancer treatment: a secondary analysis of a randomized trial. Med Decis Making. 2024;44:89–101. 37953598 10.1177/0272989X231208448 PMC10712204

[25] Kokufu H . Conflict accompanying the choice of initial treatment in breast cancer patients. Jpn J Nurs Sci. 2012;9:177–84. DOI: 10.1111/j.1742-7924.2011.00200.x 23181886

[26] Xu J Dailey RK Eggly S Neale AV Schwartz KL . Men’s perspectives on selecting their prostate cancer treatment. J Natl Med Assoc. 2011;103:468–79. 10.1016/s0027-9684(15)30359-x PMC4283563 21830629

[27] Hillen MA Gutheil CM Smets EMA , et al. The evolution of uncertainty in second opinions about prostate cancer treatment. Health Expect. 2017;20:1264–74. DOI: 10.1111/hex.12566 PMC5689232 28521078

[28] Kaplan AL Crespi CM Saucedo JD Connor SE Litwin MS Saigal CS . Decisional conflict in economically disadvantaged men with newly diagnosed prostate cancer: baseline results from a shared decision-making trial. Cancer. 2014;120:2721–7. DOI: 10.1002/cncr.28755 PMC4552329 24816472

[29] Lally RM . In the moment: women speak about surgical treatment decision making days after a breast cancer diagnosis. Oncol Nurs Forum. 2009;36(5):E257–65. 10.1188/09.ONF.E257-E265 19726385

[30] Drummond FJ Gavin AT Sharp L . Incongruence in treatment decision making is associated with lower health-related quality of life among prostate cancer survivors: results from the PiCTure study. Support Care Cancer. 2018;26:1645–54. 10.1007/s00520-017-3994-z 29222597

[31] Lantz PM Janz NK Fagerlin A , et al. Satisfaction with surgery outcomes and the decision process in a population-based sample of women with breast cancer. Health Serv Res. 2005;40:745–68. 10.1111/j.1475-6773.2005.00383.x PMC1361166 15960689

[32] Suurmond J Seeleman C . Shared decision-making in an intercultural context. Barriers in the interaction between physicians and immigrant patients. Patient Educ Couns. 2006;60:253–9. DOI: 10.1016/j.pec.2005.01.012 16442467

[33] Tong A Sainsbury P Craig J . Consolidated criteria for reporting qualitative research (COREQ): a 32-item checklist for interviews and focus groups. Int J Qual Health Care. 2007;19:349–57. DOI: 10.1093/intqhc/mzm042 17872937

[34] Straatman M. Samen beslissen bij prostaatkanker. ZorgKeuzeLab; 2017. Available from: https://zorgkeuzelab.nl/blog/Samen-beslissen-voor-betere-behandelkeuze-prostaatkanker [Accessed October, 2024].

[35] The R. Best practice: implementatie Borstkanker keuzehulp binnen OncoZON. ZorgKeuzeLab; 2017. Available from: https://zorgkeuzelab.nl/blog/samen-beslissen-bij-borstkanker [Accessed October, 2024].

[36] van Dulmen S Roodbeen R Noordman J . Tijd Voor Samen Beslissen: Perspectieven Van Patiënten, Zorgverleners En Zorgverzekeraars. Utrecht (the Netherlands): Nivel; 2020.

[37] O’Brien BC Harris IB Beckman TJ Reed DA Cook DA . Standards for reporting qualitative research: a synthesis of recommendations. Acad Med. 2014;89:1245–51. DOI: 10.1097/ACM.0000000000000388 24979285

[38] Francis JJ Johnston M Robertson C , et al. What is an adequate sample size? Operationalising data saturation for theory-based interview studies. Psychol Health. 2010;25:1229–45. 10.1080/08870440903194015 20204937

[39] Hennink M Kaiser BN . Sample sizes for saturation in qualitative research: a systematic review of empirical tests. Soc Sci Med. 2022;292:114523. DOI: 10.1016/j.socscimed.2021.114523 34785096

[40] Braun V Clarke V Hayfield N Davey L Jenkinson E . Doing reflexive thematic analysis. In: Bager-Charleson S McBeath A , eds. Supporting Research in Counselling and Psychotherapy: Qualitative, Quantitative, and Mixed Methods Research. Berlin: Springer; 2023. p 19–38.

[41] Braun V Clarke V . Using thematic analysis in psychology. Qual Res Psychol. 2006;3:77–101.

[42] Malterud K . Qualitative research: standards, challenges, and guidelines. Lancet. 2001;358:483–8. 10.1016/S0140-6736(01)05627-6 11513933

[43] van Tilburg-Huisman C Twisk M Stibbe K Nieuwenhuyzen-de Boer G Aarts A . Samen beslissen: bewustwording is het halve werk. *Nederlands Tijdschrift voor Obstetrie & Gynaecoloige*. 2021;134(6):280–282

[44] Chewning B Bylund CL Shah B Arora NK Gueguen JA Makoul G . Patient preferences for shared decisions: a systematic review. Patient Educ Couns. 2012;86:9–18. 21474265 10.1016/j.pec.2011.02.004 PMC4530615

[45] Keij SM van Duijn-Bakker N Stiggelbout AM Pieterse AH . What makes a patient ready for shared decision making? A qualitative study. Patient Educ Couns. 2021;104:571–7. DOI: 10.1016/j.pec.2020.08.031 32962880

[46] Keij SM Stiggelbout AM Pieterse AH . Patient readiness for shared decision making about treatment: conceptualisation and development of the Ready(SDM). Health Expect. 2024;27:e13995. DOI: 10.1111/hex.13995 PMC10891436 38400633

[47] Joseph-Williams N Elwyn G Edwards A . Knowledge is not power for patients: a systematic review and thematic synthesis of patient-reported barriers and facilitators to shared decision making. Patient Educ Couns. 2014;94:291–309. 24305642 10.1016/j.pec.2013.10.031

[48] Muscat DM Shepherd HL Nutbeam D Trevena L McCaffery KJ . Health literacy and shared decision-making: exploring the relationship to enable meaningful patient engagement in healthcare. J Gen Intern Med. 2021;36:521–4. DOI: 10.1007/s11606-020-05912-0 PMC7878628 32472490

[49] Thunholm P . Decision-making styles and physiological correlates of negative stress: is there a relation? Scand J Psychol. 2008;49:213–9. DOI: 10.1111/j.1467-9450.2008.00640.x 18384496

[50] Scott SG Bruce RA . Decision-making style: the development and assessment of a new measure. Educ Psychol Meas. 1995;55:818–31.

[51] Leykin Y DeRubeis RJ . Decision-making styles and depressive symptomatology: development of the decision styles questionnaire. Judgm Decis Mak. 2010;5:506–15.

[52] Bélanger E Rodríguez C Groleau D Légaré F MacDonald ME Marchand R . Patient participation in palliative care decisions: an ethnographic discourse analysis. Int J Qual Stud Health Well-Being. 2016;11:32438. 27882864 10.3402/qhw.v11.32438 PMC5122231

[53] Emanuel EJ Emanuel LL . Four models of the physician-patient relationship. JAMA. 1992;267:2221–6. 1556799

[54] Kon AA . The shared decision-making continuum. JAMA. 2010;304:903–4. 10.1001/jama.2010.1208 20736477

[55] MacArtney JI Andersen RS Malmstrom M Rasmussen B Ziebland S . The convivial and the pastoral in patient-doctor relationships: a multi-country study of patient stories of care, choice and medical authority in cancer diagnostic processes. Sociol Health Illn. 2020;42:844–61. DOI: 10.1111/1467-9566.13067 PMC7318254 32103515

[56] Pilnick A . Reconsidering patient-centred care: authority, expertise and abandonment. Health Expect. 2023;26(5):1785–8. 10.1111/hex.13815 PMC10485311 37469280

[57] Ubel PA Scherr KA Fagerlin A . Autonomy: what’s shared decision making have to do with it? Am J Bioeth. 2018;18:W11–2. DOI: 10.1080/15265161.2017.1409844 PMC6312719 29393797

[58] Rollnick S Miller WR Butler CC . Motivational Interviewing in Health Care: Helping Patients Change Behavior. London: Taylor & Francis; 2008.

[59] Moon MD . Triangulation: a method to increase validity, reliability, and legitimation in clinical research. J Emerg Nurs. 2019;45:103–5. 10.1016/j.jen.2018.11.004 30616761

[60] Denzin NK . Sociological Methods: A Sourcebook. London: Routledge; 2017.

