# Supplementary material for: Cancer Patients’ Experiences of Burden when Involved in Treatment Decision Making
Source: Med Decis Making. 2025 Apr 29;45(5):533–44. doi: 10.1177/0272989X251334979 (PMC12166153; doi:10.1177/0272989X251334979)
Supplement: sj-docx-1-mdm-10.1177_0272989X251334979 – Supplemental material for Cancer Patients’ Experiences of Burden when Involved in Treatment Decision Making [file sj-docx-1-mdm-10.1177_0272989X251334979.docx]

**Appendices**

**Appendix 1. COREQ checklist**

Consolidated criteria for reporting qualitative studies (COREQ): 32-item checklist

| **No. Item** | **Guide questions/description** | **Reported on Page #** |
| --- | --- | --- |
| **Domain 1: Research team and reﬂexivity** |  |  |
| *Personal Characteristics* |  |  |
| 1. Inter viewer/facilitator | Which author/s conducted the inter view or focus group? | Page 5, line 114-117 |
| 2. Credentials | What were the researcher’s credentials? E.g. PhD, MD | Page 1  Page 5, line 114-117 |
| 3. Occupation | What was their occupation at the time of the study? | Page 5, line 114-117 |
| 4. Gender | Was the researcher male or female? | n/a |
| 5. Experience and training | What experience or training did the researcher have? | Page 5, line 114-117 |
| *Relationship with participants* |  |  |
| 6. Relationship established | Was a relationship established prior to study commencement? | n/a |
| 7. Participant knowledge of the interviewer | What did the participants know about the researcher? e.g. personal goals, reasons for doing the research | n/a |
| 8. Interviewer characteristics | What characteristics were reported about the inter viewer/facilitator? e.g. Bias, assumptions, reasons and interests in the research topic | Page 5, line 114-121 |
| **Domain 2: study design** |  |  |
| *Theoretical framework* |  |  |
| 9. Methodological orientation and Theory | What methodological orientation was stated to underpin the study? e.g. grounded theory, discourse analysis, ethnography, phenomenology, content analysis | Page 6, line 127-129 |
| *Participant selection* |  |  |
| 10. Sampling | How were participants selected? e.g. purposive, convenience, consecutive, snowball | Page 5, line 99-105 |
| 11. Method of approach | How were participants approached? e.g. face-to-face, telephone, mail, email | Page 5, line 101-108 |
| 12. Sample size | How many participants were in the study? | Page 6, line 135 |
| 13. Non-participation | How many people refused to participate or dropped out? Reasons? | Page 6, line 134-136 |
| *Setting* |  |  |
| 14. Setting of data collection | Where was the data collected? e.g. home, clinic, workplace | Page 5, line 108-109 |
| 15. Presence of non-participants | Was anyone else present besides the participants and researchers? | n/a |
| 16. Description of sample | What are the important characteristics of the sample? e.g. demographic data, date | Page 6, line 134-140 |
| *Data collection* |  |  |
| 17. Interview guide | Were questions, prompts, guides provided by the authors? Was it pilot tested? | Page 5&6, line 118-125 |
| 18. Repeat interviews | Were repeat inter views carried out? If yes, how many? | n/a |
| 19. Audio/visual recording | Did the research use audio or visual recording to collect the data? | Page 6, line 125 |
| 20. Field notes | Were ﬁeld notes made during and/or after the inter view or focus group? | Page 5, line 119 -121 |
| 21. Duration | What was the duration of the inter views or focus group? | Page 6, line 125-126 |
| 22. Data saturation | Was data saturation discussed? | Page 5, line 110-112 |
| 23. Transcripts returned | Were transcripts returned to participants for comment and/or correction? | Figure 1 |
| **Domain 3: analysis and ﬁndings** |  |  |
| *Data analysis* |  |  |
| 24. Number of data coders | How many data coders coded the data? | Figure 1 |
| 25. Description of the coding tree | Did authors provide a description of the coding tree? | n/a |
| 26. Derivation of themes | Were themes identiﬁed in advance or derived from the data? | Page 6, line 128-129  Figure 1 |
| 27. Software | What software, if applicable, was used to manage the data? | Page 6, line 130 |
| 28. Participant checking | Did participants provide feedback on the ﬁndings? | Figure 1 |
| *Reporting* |  |  |
| 29. Quotations presented | Were participant quotations presented to illustrate the themes/ﬁndings? Was each quotation identiﬁed? e.g. participant number | Table 3 |
| 30. Data and ﬁndings consistent | Was there consistency between the data presented and the ﬁndings? | Page 6 -10 |
| 31. Clarity of major themes | Were major themes clearly presented in the ﬁndings? | Figure 2 |
| 32. Clarity of minor themes | Is there a description of diverse cases or discussion of minor themes? | Page 6 -10 |

**Appendix 2. Screening questionnaire**

**Question 1. Diagnosis**
Have you been diagnosed with early-stage breast or prostate cancer within the past 6 months? By early stage we mean you have no metastases and the cancer is curable.

- Yes
- No

**Question 2. Age**What age group are you in? We ask this because we want to interview a diverse group of patients.

- Under 45 years of age
- 45-59 years
- 60-74 years
- 75 years or over

**Question 3. Choice of treatment**After a cancer diagnosis, people talk to their doctor about treatment. Sometimes it is possible to wait with treatment or not to treat at all. And sometimes several types of treatment are possible. In all cases, there is a choice. Have you ever had such a choice of treatment options?

- Yes
- No

If so, what did this choice consist of? Explain in one or two sentences.

………………………………………………………………………………………………………………………………………………………………………………………………………………………………………………………………………………………………………………………….

If not, why was there no choice? Explain in one or two sentences.

*For example: because no choice was medically possible or because the doctor did not present a choice to you.*

………………………………………………………………………………………………………………………………………………………………………………………………………………………………………………………………………………………………………………………….

**Question 4. Burden regarding treatment choice**When thinking about the treatment choice you found most difficult. How difficult was making this choice for you?

*‼ Choose one of the following answers.*

- Not at all burdensome
- Not burdensome
- Somewhat burdensome
- Burdensome
- Very much burdensome

**Appendix 3. Interview guide**

**Interview study with patients (ParadoX of Choice)**

**Aim:** To describe the nature and origins of the burden of shared decision making in cancer care. This study examines how patients are burdened by shared decision making, to what extent, and in which situations. This study will describe how patients experience and explain the burden and what they believe is helpful in mitigating the burden.

Research question:

What is the nature of the burden of shared decision making and how do patients explain it?

Sub-questions:

1) How do patients reflect on the manner and extent in which they were involved in decision making about their treatment?

2) How did patients’ involvement affected them positively and negatively?

3) What do patients believe could reduce the negative effects of their involvement?

**Semi-structured interview guide**

**Description of the situation**

- Medical background
  - What is your diagnosis? When were you diagnosed? Where are you in the care process now?
- You have indicated that at some point there was a choice between different treatment options. Can you take me back to the moment that you were presented with this choice by the clinician?
  - What were the treatment options?
- When you heard from the doctor what the treatment options were, how did you feel about it?

**Experienced of burden**

- You indicated in the questionnaire that choice X was somewhat difficult. Could you tell me in your own words what you found difficult?
  - What made this so difficult for you?
  - How exactly did this burden manifest itself? For example, did you sleep worse because of it? Did you experience certain emotions?
- What was it like for the other choices? Were they equally difficult? Why/why not?
  - What made that choice less difficult?/Or were those choices difficult for a different reason?
- How uncertain were you during this period/about making a choice? / What role did uncertainty play in making a choice?
  - How did you deal with these feelings?
- You indicated that you found the decision difficult at the time, but what is it like for you at this moment? How do you feel now, a few months later after making this choice?
  - How did it feel when the decision was made?
  - Do you look back on it differently now than at the time of/shortly after the decision?

**Mitigating burden**

- What would have helped to make the choice easier/less burdensome? What was already going well, what could be done differently?
- What would you like to see different regarding the decision making process for future patients who find themselves in the same situation as you?

**Closing**

- What else would you like to say about your involvement in treatment decision making and the burden that came/comes with it for you as a patient?
